# Time-Varying Channel Estimation Based on Distributed Compressed Sensing for OFDM Systems

**DOI:** 10.3390/s24113581

**Published:** 2024-06-01

**Authors:** Yong Ding, Honggao Deng, Yuelei Xie, Haitao Wang, Shaoshuai Sun

**Affiliations:** 1 School of Information and Communication, Guilin University of Electronic Technology, Guilin 541004, China; ding_yong@guet.edu.cn (Y.D.); ylxie@guet.edu.cn (Y.X.); wanghaitao@mails.guet.edu.cn (H.W.); sunss@guet.edu.cn (S.S.); 2 State and Local Joint Engineering Research Center for Satellite Navigation and Location Service, Guilin University of Electronic Technology, Guilin 541004, China

**Keywords:** orthogonal frequency division multiplexing (OFDM), time-varying channel estimation, basis expansion model (BEM), distributed compressed sensing (DCS), symmetric extension

## Abstract

For orthogonal frequency division multiplexing (OFDM) systems in high-mobility scenarios, the estimation of time-varying multipath channels not only has a large error, which affects system performance, but also requires plenty of pilots, resulting in low spectral efficiency. To address these issues, we propose a time-varying multipath channel estimation method based on distributed compressed sensing and a multi-symbol complex exponential basis expansion model (MS-CE-BEM) by exploiting the temporal correlation and the joint delay sparsity of wideband wireless channels within the duration of multiple OFDM symbols. Furthermore, in the proposed method, a sparse pilot pattern with the self-cancellation of pilot intercarrier interference (ICI) is adopted to reduce the input parameter error of the MS-CE-BEM, and a symmetrical extension technique is introduced to reduce the modeling error. Simulation results show that, compared with existing methods, this proposed method has superior performances in channel estimation and spectrum utilization for sparse time-varying channels.

## 1. Introduction

With the rapid development of transportation modes, such as high-speed railways, highways, and unmanned aerial vehicles (UAVs), etc., the integration of wideband wireless communication in these high-mobility scenarios into novel networks has been a research topic in recent years [1,2,3]. According to the 5G standard [4] and the future 6G vision, novel networks must provide a higher data rate, lower latency, better reliability, wider coverage, low power consumption, and low-cost communication services. Non-orthogonal multiple access (NOMA), massive multiple-input multiple-output (MIMO), mmWave, mobile edge computing (MEC), and artificial intelligence (AI) are the promising technologies that meet these requirements [5]. Specifically, orthogonal time-frequency-space (OTFS) is a recently proposed modulation technique for high-mobility communication. In contrast to orthogonal frequency division multiplexing (OFDM), which modulates information in the time-frequency domain, OTFS modulates information in the delay-Doppler domain, and provides strong delay and Doppler adaptability, possessing the potential of full diversity [6]. However, as a nascent modulation technology, OTFS faces challenges in compatibility, complexity, and standardization, compared to more mature OFDM. In particular, OTFS may have some difficulties in achieving efficient performance in multipath fast-varying channels [7].

The application of OFDM in high-mobility scenarios still attracts great attention. However, the multipath channels in these scenarios experience rapid fluctuations, with the Doppler spread destroying the orthogonality among OFDM subcarriers, ultimately leading to intercarrier interference (ICI). In particular, as mobility increases, the channel taps undergo significant changes within an OFDM symbol, making ICI non-negligible. If ICI is not effectively suppressed, it can seriously degrade the system’s performance [3].

There are several categories of methods to suppress ICI. The first category of methods suppresses the ICI on all subcarriers by ICI self-cancellation [8] and adding virtual carriers (VCs) [7]. This category of methods has low complexity but low spectral efficiency. The second category of methods begins by modeling time-varying channel taps. Subsequently, they estimate the time-varying channel using model coefficients. Finally, they suppress ICI by equalizing the received symbols in the frequency domain. The third category of methods estimates channels based on decision feedback, which reduces the impact of ICI and additive noise through iterative processes [9]. The fourth category of time-varying channel estimation methods is based on deep learning and has emerged in recent years [10]. The latter two categories of methods assume that the channel taps remain basically unchanged or change very little within an OFDM symbol. In addition, due to the low carrier frequency of underwater communication systems, the impact of ICI on their performance is non-negligible [11], and the ICI suppression techniques employed in these systems can serve as a reference.

In the second category of methods mentioned above, several models are used to model time-varying channels, among which the complex exponential basis expansion model (CE-BEM) [12] is widely utilized due to its independence from channel statistics. However, when the CE-BEM is adopted, a large number of pilot subcarriers are still needed to estimate BEM coefficients, resulting in low spectral efficiency. In addition, Gibbs phenomenon in the CE-BEM can increase the model error, and the ICI on the pilots can reduce the estimation accuracy of the BEM coefficients. Both factors can lead to a decrease in channel estimation performance.

The temporal correlation and delay sparsity of channels provide some ways to address the above issues. Research in [13,14,15] indicates that, despite the rapid changes in time-varying channels over time, they still exhibit a temporal correlation, hence path delays can remain relatively constant in multiple OFDM symbols. By using this characteristic of time-varying channels, the joint channel estimation within multiple OFDM symbols can improve the estimation performance compared with that within a single OFDM symbol [15,16]. In [16], we proposed a channel estimation method based on the multi-symbol CE-BEM (MS-CE-BEM), which achieves a reduction in pilots and an improvement of the estimation performance. On the other hand, many wideband wireless channels exhibit delay sparsity [17,18]. In [19,20], the compressed sensing (CS) theory was applied to the estimation of sparse channels in OFDM systems, resulting in a reduction in pilots and an improvement of the estimation performance. As an extension of CS, the distributed compressed sensing (DCS) method [21,22] can jointly reconstruct multiple sparse signals. It is proved in [23] that the DCS-based OFDM channel estimation method has a better performance than the CS-based one. However, this method only exploits channel sparsity within a single OFDM symbol.

To address the two issues of large channel estimation errors and low spectrum utilization of OFDM systems in high-mobility scenarios, we propose a time-varying channel estimation method in this paper based on DCS and the MS-CE-BEM. The main contribution of this paper is three-fold:

- On the basis of the previously proposed MS-CE-BEM, to reduce its model error, we extend the symmetric extension technique in [24] to multiple OFDM symbols for mitigating the Gibbs phenomenon.
- Exploiting the temporal correlation and the joint sparsity of wideband wireless channels within multiple OFDM symbols, we construct a DCS framework to estimate the time average of the tap gain (TATG) matrix for multiple OFDM symbols, which is used as the input parameter matrices of the MS-CE-BEM.
- To reduce the impact of pilot ICI on the estimation of the TATG matrix, we adopt a sparse pilot pattern with pilot ICI self-cancellation. Meanwhile, the placement of pilot clusters in the pilot pattern is optimized to ensure that the TATG matrix can be reconstructed with high probability under the DCS framework.

Compared with the method in [16], the proposed method not only has a higher channel estimation performance, but also saves a large number of pilots, resulting in the improvement of spectral efficiency. This is because we utilize the temporal correlation and the joint sparsity of the channel within multiple OFDM symbols.

The remainder of the paper is organized as follows. Section 2 introduces the OFDM system model. In Section 3, we present the channel estimation model (MS-CE-BEM) that incorporates the symmetric extension technique. The DCS-based estimation for the input parameter matrix of the MS-CE-BEM is addressed in Section 4. The channel estimation process of the proposed method is summarized in Section 5. Simulation results are provided in Section 6 to demonstrate the superior performance of the proposed method. Finally, Section 7 concludes the paper.

The notations adopted are as follows. Upper (lower) boldface letters denote matrices (column vectors). ${\left[x\right]}_{k}$ denotes the *k*-th element of the vector x, and ${\left[X\right]}_{k,m}$ denotes the $\left[k,m\right]$-th element of matrix X. $I_{k}$ and $0_{k}$ denote the k×k identity matrix and k×k all-zero matrix, respectively. $X^{\left(\mathrm{flip}\right)}$ denotes the matrix obtained by flipping the columns of matrix X around the vertical axis. diag{x} denotes a diagonal matrix with x on its main diagonal. Tr(⋅), ${\left(\cdot \right)}^{T}$, ${\left(\cdot \right)}^{H}$, and ${\left(\cdot \right)}^{†}$ represent trace, transpose, Hermitian, and pseudo-inverse operators, respectively. $\langle x,y\rangle$ denotes the inner product of the vectors x and y, and $||x|{|}_{2}$ denotes the 2-norm of the vector x. E{⋅}, ${((\cdot ))}_{N}$, and $\left\lceil \cdot \right\rceil$ denote expectation, modulo-*N*, and upper rounding operators, respectively. $\left|x\right|$ denotes the magnitude of the variable x, and |P| denotes the cardinality of the set P. In addition, all the subscripts and indices in this paper begin with zero.

## 2. OFDM System Model

We consider an OFDM system with *N* subcarriers. At the transmitter, the *m*-th OFDM frequency domain symbols $x^{\left(m\right)}\in {\mathbb{C}}^{N\times 1}$(0≤m≤M−1) are transformed into the time domain samples by an *N*-point inverse discrete Fourier transform (IDFT). After inserting a cyclic prefix (CP) of length *N*g, the time domain signal is transmitted through a time-varying multipath channel with a maximum Doppler frequency, *f*_d_. Assuming that the system sampling period is *T*_s_, the gain of the *l*-th channel tap at time *nT*_s_ is denoted as $h_{n,l}$, 0≤l≤L−1. *L* is the total number of channel taps, and $L\leq N_{g}$.

At the receiver, after removing the CP and performing an *N*-point discrete Fourier transform (DFT), the corresponding frequency domain-received symbols can be obtained as

(1) $$y^{\left(m\right)}=H_{F}^{\left(m\right)}x^{\left(m\right)}+w^{\left(m\right)}$$

where $w^{\left(m\right)}\in {\mathbb{C}}^{N\times 1}$ is the frequency domain representation of the complex additive white Gaussian noise (AWGN) and $H_{F}^{\left(m\right)}$ is the channel frequency response (CFR) matrix. $H_{F}^{\left(m\right)}$ can be expressed as

(2) $$H_{F}^{\left(m\right)}=\frac{1}{N}FH_{T}^{\left(m\right)}F^{H}$$

where $F\in {\mathbb{C}}^{N\times N}$ is the DFT matrix whose elements are ${\left[F\right]}_{u,v}=e^{-j\frac{2\pi }{N}uv}$, 0≤u,v≤N−1, and $H_{T}^{\left(m\right)}\in {\mathbb{C}}^{N\times N}$ is the channel impulse response (CIR) matrix whose elements are ${\left[H_{T}^{\left(m\right)}\right]}_{u,v}=h_{mN_{v}+u,{\left((u-v)\right)}_{N}}$, 0≤u,v≤N−1. Here, $N_{v}$ denotes the length of an OFDM symbol with CP, i.e., $N_{v}=N+N_{g}$. The diagonal elements of $H_{F}^{\left(m\right)}$ are ${\left[H_{F}^{\left(m\right)}\right]}_{u,u}=\sum_{l=0}^{L-1}\alpha_{l}^{\left(m\right)}e^{-j2\pi \frac{u}{N}l}$, where $\alpha_{l}^{\left(m\right)}$ is the TATG during the effective duration of the *m*-th OFDM symbol, expressed as

(3) $$\alpha_{l}^{\left(m\right)}=(1/N)\sum_{n=0}^{N-1}h_{mN_{v}+n,l}.$$

where $T=N\cdot T_{s}$ denotes the duration of an OFDM symbol without a CP, *f*_d_*T* is the normalized Doppler frequency that describes the rate of channel change. For a static channel, $H_{F}^{\left(m\right)}$ is a diagonal matrix. With the increase in *f*_d_*T*, however, $h_{n,l}$ changes significantly within an OFDM symbol, and the off-diagonal elements of $H_{F}^{\left(m\right)}$ are not negligible [12]. Consequently, Equation (1) can be rewritten as

(4) $$y^{\left(m\right)}=\mathrm{diag}\{x^{\left(m\right)}\}F_{L}\alpha^{\left(m\right)}+{\bar{H}}_{F}^{\left(m\right)}x^{\left(m\right)}+w^{\left(m\right)}$$

where $F_{L}\in {\mathbb{C}}^{N\times L}$ denotes the submatrix extracting the first *L* columns of F, ${\bar{H}}_{F}^{\left(m\right)}$ denotes the matrix obtained by setting the diagonal elements of $H_{F}^{\left(m\right)}$ to zero, and $\alpha^{\left(m\right)}={\left[\alpha_{0}^{\left(m\right)},\;\ldots ,\;\alpha_{L-1}^{\left(m\right)}\right]}^{T}$.

The TATG matrix of *M* consecutive OFDM symbols is denoted as $A\in {\mathbb{C}}^{L\times M}$, where the *l*-th row of A corresponds to *l*-th channel tap TATGs within each individual OFDM symbol, denoted as $\alpha_{l}={\left[\alpha_{l}^{\left(0\right)},\;\ldots ,\;\alpha_{l}^{\left(M-1\right)}\right]}^{T}$, and the *m*-th column of A corresponds to the TATGs of each individual channel tap within the *m*-th OFDM symbol, i.e., $\alpha^{\left(m\right)}$. We discuss in the next section that the TATG matrix, A, is the input parameter matrix of the proposed MS-CE-BEM.

## 3. MS-CE-BEM’s Modeling of Channels

In this section, we first explore the temporal correlation of time-varying channels, which forms the foundation for the MS-CE-BEM. Subsequently, we introduce the MS-CE-BEM with the symmetric extension technique that can reduce model errors. Finally, we examine the upper limit of *f*_d_*T* for the time-varying channels to which the proposed channel estimation model can be applied. The existence of the upper limit of *f*_d_*T* is a limiting condition for the application of the proposed model.

### 3.1. Temporal Correlation of Time-Varying Channels

For time-varying multipath channels, the path delays usually vary much slower than the path gains, and thus the channels exhibit a temporal correlation. Even for high-mobility scenarios, the path delays may remain relatively unchanged within multiple OFDM symbols, although the path gains change within one symbol [13,14]. According to [15], it can be assumed that the nonzero element positions of the CIR remain unchanged within *M* consecutive OFDM symbols, as long as $M<\frac{0.01c}{(N+N_{g})v}$ is satisfied, where *c* is the speed of light and *v* is the speed of the receiver relative to the transmitter. For example, the maximum value of M is 27 when N=1024, $N_{g}=64$, and v=360 km/h.

### 3.2. MS-CE-BEM Incorporating Symmetric Extension

**Figure 1:**
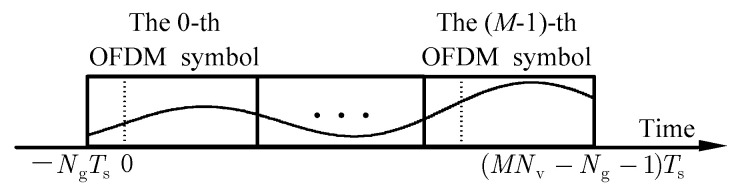
Variation in real or imaginary parts of a channel tap within *M* consecutive OFDM symbols.

Utilizing the temporal correlation of time-varying channels, the multi-symbol BEM method [16] treats *M* consecutive OFDM symbols as a whole for joint channel estimation. During this period, the gain vector of the *l*-th channel tap is denoted as $h_{l}={\left[h_{-N_{g},l},\;\ldots ,\;h_{N_{v}M-N_{g}-1,l}\right]}^{T}\in {\mathbb{C}}^{MN_{v}\times 1}$, as shown in Figure 1.

Correspondingly, the gain vector of the *l*-th channel tap during the effective duration of the *m*-th OFDM symbol can be expressed as

(5) $$h_{l}^{\left(m\right)}=Q^{\left(m\right)}h_{l},\;0\leq m\leq M-1$$

where $Q^{\left(m\right)}\in {\mathbb{R}}^{N\times MN_{v}}$ is the segment selection matrix of channel taps, given by

(6) $$Q^{\left(m\right)}=\underset{(mN_{v}+N_{g})\;\mathrm{columns}}{\underset{︸}{\left[\begin{array}{ccc} 0 & \cdots & 0\\ 0 & \cdots & 0\\ \vdots & \vdots & \vdots \\ 0 & \cdots & 0 \end{array}\right.}}\;\underset{N\;\mathrm{columns}}{\underset{︸}{\begin{array}{cccc} 1 & 0 & \cdots & 0\\ 0 & 1 & \cdots & 0\\ \vdots & \vdots & \ddots & \vdots \\ 0 & 0 & \cdots & 1 \end{array}}}\;\underset{((M-m-1)N_{v})\;\mathrm{columns}}{\underset{︸}{\left.\begin{array}{ccc} 0 & \cdots & 0\\ 0 & \cdots & 0\\ \vdots & \vdots & \vdots \\ 0 & \cdots & 0 \end{array}\right]}}$$

**Figure 2:**
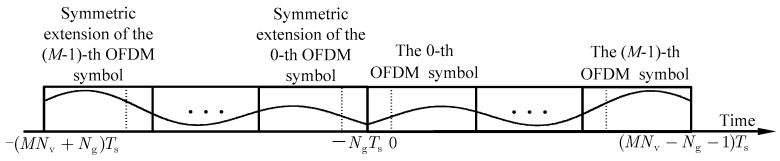
Schematic diagram for symmetric expansion.

Since the channel tap gains are frequently unequal at both ends of the duration, there is the Gibbs phenomenon in the CE-BEM expansion of the channel tap, resulting in a high model error. Inspired by [24], we utilize the symmetric expansion technique to suppress the Gibbs phenomenon caused by the MS-CE-BEM, which is different from the baseline tilting technique in [16]. Figure 2 shows the schematic diagram of symmetric expansion.

The symmetric extended gain vector of the *l*-th channel tap can be expressed as ${h^{\prime }}_{l}=U_{MN_{v}}h_{l}$, where $U_{MN_{v}}={\left[I_{MN_{v}}^{\left(\mathrm{flip}\right)},I_{MN_{v}}\right]}^{T}$ is the symmetric extension matrix of channel taps. Accordingly, $h_{l}$ can be expressed by ${h^{\prime }}_{l}$ as

(7) $$h_{l}=U_{MN_{v}}^{(c)}{h^{\prime }}_{l}$$

where $U_{MN_{v}}^{(c)}=[0_{MN_{v}},I_{MN_{v}}]$. Expanding ${h^{\prime }}_{l}$ with the BEM, we can obtain

(8) $${h^{\prime }}_{l}=Bg_{l}+\xi_{l}$$

where $B=[b_{0},\;\cdots ,\;b_{Q}]\in {\mathbb{C}}^{2MN_{v}\times (Q+1)}$, $g_{l}={\left[g_{0,l},\;\cdots ,\;g_{Q,l}\right]}^{T}\in {\mathbb{C}}^{(Q+1)\times 1}$, and $\xi_{l}\in {\mathbb{C}}^{2MN_{v}\times 1}$ are the basis function matrix, the BEM coefficient vector, and the model error vector, respectively. The basis vectors $b_{q}$(q=0,…,Q) in B can denoted as $b_{q}={\left[b_{q,-N_{v}M-N_{g}},\;\cdots ,\;b_{q,-N_{g}},\;\cdots ,\;b_{q,N_{v}M-N_{g}-1}\right]}^{T}$, where $b_{q,n}=e^{j2\pi (q-Q/2)n/(2MN_{v})}$ for the CE-BEM. Obviously, ${h^{\prime }}_{l}$ has equal values at both ends, and therefore its CE-BEM expansion error is smaller [24]. The symmetric expansion of the vector $\alpha_{l}={\left[\alpha_{l}^{\left(0\right)},\;\ldots ,\;\alpha_{l}^{\left(M-1\right)}\right]}^{T}$ can be expressed as ${\alpha^{\prime }}_{l}=U_{M}\alpha_{l}$, where $U_{M}={\left[I_{M}^{\left(\mathrm{flip}\right)},I_{M}\right]}^{T}$ is the TATG symmetric extension matrix. On the other hand, according to Equation (8), ${\alpha^{\prime }}_{l}$ can also be expressed as

(9) $${\alpha^{\prime }}_{l}=D{h^{\prime }}_{l}=DBg_{l}+D\xi_{l}$$

where $D\in {\mathbb{R}}^{2M\times 2MN_{v}}$ is the transfer matrix between ${h^{\prime }}_{l}$ and ${\alpha^{\prime }}_{l}$. And D can be expressed as

(10) $$D=\left[\begin{array}{cc} D_{1} & \;\\ \; & D_{2} \end{array}\right]$$

where the submatrices $D_{1},D_{2}\in {\mathbb{R}}^{M\times MN_{v}}$ are given by

(11) D1=1N⋯1N0⋯0⋮⋮⋮0⋯0︸N columns 0⋯00⋯0⋮⋮⋮0⋯0︸Ng columns︸Symmetric symbol of the (M−1)-th OFDM symbol ⋯⋯⋮⋯ 0⋯00⋯0⋮⋮⋮1N⋯1N︸N columns 0⋯00⋯0⋮⋮⋮0⋯0︸Ng columns︸Symmetric symbol of the 0-th OFDM symbol

(12) D2=0⋯00⋯0⋮⋮⋮0⋯0︸Ng columns 1N⋯1N0⋯0⋮⋮⋮0⋯0︸N columns︸The 0-th OFDM symbol  ⋯⋯⋮⋯ 0⋯00⋯0⋮⋮⋮0⋯0︸Ng columns 0⋯00⋯0⋮⋮⋮1N⋯1N︸N columns︸the (M−1)-th OFDM symbol.

${\left[D_{1}\right]}_{m,n_{1}:n_{2}}$ and ${\left[D_{2}\right]}_{m,n_{1}:n_{2}}$ are used to denote the elements in the *m*-th rows of $D_{1}$ and $D_{2}$ from column $n_{1}$ to column $n_{2}$, respectively. And then [D1]m,mNv:(m+1)Nv−Ng−1=1N and [D2]m,mNv+Ng:((m+1)Nv−1)=1N for 0≤m≤M−1. Apart from the elements mentioned above, all other elements in $D_{1}$ and $D_{2}$ are zero.

When 2M≥(Q+1), by utilizing the least squares (LS) algorithm, the approximation of $g_{l}$ in Equation (9) can be obtained as

(13) $${\tilde{g}}_{l}={\left(DB\right)}^{†}{\alpha^{\prime }}_{l}={\left(DB\right)}^{†}U_{M}\alpha_{l}.$$

Furthermore, from Equations (5), (7), (8), and (13), the fitted value of $h_{l}^{\left(m\right)}$ can be obtained as

(14) $${\tilde{h}}_{l}^{\left(m\right)}=Q^{\left(m\right)}U_{MN_{v}}^{(c)}B{\tilde{g}}_{l}=Q_{m}U_{MN_{v}}^{(c)}B{\left(DB\right)}^{†}U_{M}\alpha_{l}=V^{\left(m\right)}\alpha_{l}$$

where $V^{\left(m\right)}\in {\mathbb{C}}^{N\times M}$ is the transformation matrix between ${\tilde{h}}_{l}^{\left(m\right)}$ and $\alpha_{l}$, and given by

(15) $$V^{\left(m\right)}=Q^{\left(m\right)}U_{MN_{v}}^{(c)}B{\left(DB\right)}^{†}U_{M}.$$

As long as the receiver obtains the estimation of $\alpha_{l}$, the estimation of $h_{l}^{\left(m\right)}$ can be obtained according to Equation (14). The CIR matrix, $H_{T}^{\left(m\right)}$, can be constructed from $h_{l}^{\left(m\right)},0\leq l\leq L-1$. Subsequently, according to Equation (2), the CFR matrix, $H_{F}^{\left(m\right)}$, can be calculated for the frequency domain equalization of the *m*-th OFDM symbol. Thus, the TATG matrix, $A=[\alpha_{0},\ldots ,\alpha_{L-1}{]}^{T}\in {\mathbb{C}}^{L\times M}$, can be considered as the input parameter matrix of the MS-CE-BEM. In Section 4, we will estimate A in the DCS framework.

### 3.3. Upper Limit of f_*d*_T

In order to approximate time-varying channels effectively, the number of basis vectors of the CE-BEM needs to satisfy $Q\geq 2\left\lceil 2f_{d}T_{s}(N+N_{g})M\right\rceil$ according to [12]. Incorporating 2M≥(Q+1), we can obtain

(16) $$f_{d}T\leq \frac{(M-1)N}{2M(N+N_{g})}.$$

Equation (16) shows that *f*_d_*T* has an upper limit in the MS-CE-BEM with the symmetric extension technique. When *f*_d_*T* is greater than its upper limit, the MS-CE-BEM cannot effectively approximate the time variation in channel taps, resulting in a rapid decline in channel estimation performance. The existence of the upper limit of *f*_d_*T* represents a limitation in the application of the MS-CE-BEM. The proposed method is based on the MS-CE-BEM, and therefore cannot overcome this limitation.

**Figure 3:**
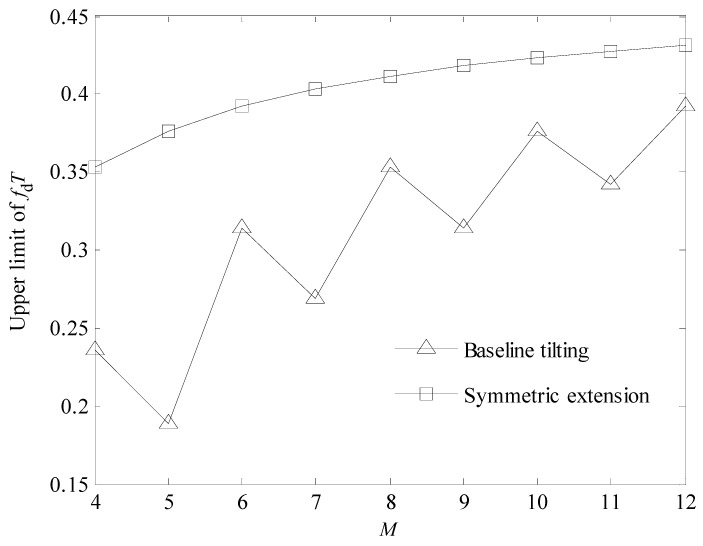
Upper limits of *f*_d_*T* corresponding to the two techniques for suppressing the Gibbs phenomenon.

In the MS-CE-BEM, for different *M* values, the upper limits of *f*_d_*T* corresponding to the symmetric extension technique and the baseline tilting technique [16] are shown in Figure 3. In the calculations, the number of subcarriers is N=1024 and the CP length is $N_{g}=64$.

From Figure 3, it can be observed that the upper limit of *f*_d_*T* of the symmetric expansion technique is higher than that of the baseline tilting technique. Therefore, it can be applied to faster time-varying channels. This is because the baseline tilting technique needs an extra basis vector to eliminate the difference at both ends of the channel tap, and its capability to approximate time variation is inferior to that of the symmetric expansion technique.

Comparative analysis confirms that the proposed model incorporating the symmetric extension technique is superior to the one incorporating the baseline tilting technique in terms of the upper limit of *f*_d_*T*.

## 4. DCS-Based Estimation of the TATG Matrix

In this section, we estimate the input parameter matrix of the MS-CE-BEM, i.e., A, in the DCS framework. For DCS, consider *M* CS problems $z^{\left(m\right)}=\Phi \alpha^{\left(m\right)}+\gamma^{\left(m\right)}$, m∈{0,1,…,M−1}. Suppose that each vector, $\alpha^{\left(m\right)}$, has *K* nonzero elements and appears in the same position, that is, the sparse vector set $\left\{\alpha^{\left(m\right)}\right\}$ satisfies the joint sparsity model-2 (JSM-2) proposed in [21]. Unlike CS, which reconstructs $\alpha^{\left(m\right)}$ individually, DCS aims to jointly reconstruct the sparse vector set $\left\{\alpha^{\left(m\right)}\right\}$, using the common measurement matrix Φ. As is well known, CS utilizes the inherent sparsity of sparse signals. On the basis of CS, DCS further leverages the correlation among multiple sparse signals. By jointly processing multiple sparse signals, DC can search for the correct positions of their nonzero elements with a higher probability, thereby exhibiting significant advantages in terms of recovery accuracy [22].

In this section, we first discuss the joint sparsity of the TATG matrix in wideband wireless systems. Then, the DCS framework for estimating the TATG matrix is constructed based on the pilot pattern with pilot ICI self-cancellation. Finally, the placement of pilot clusters is optimized.

### 4.1. Joint Sparsity of the TATG Matrix in Wideband OFDM Systems

In wideband OFDM systems, the delay intervals of physical paths are frequently far greater than the sampling period, $T_{s}$, which means that many channel taps may not contain physical paths. Thus, the corresponding channels exhibit sparsity in the delay domain [17,18]. Here, we introduce the definition of the *K*-sparse channel based on [15].

**Definition** **1.** *Let* $K_{n}=\{l|\left|h_{n,l}\right|>\varepsilon \}$ *denote the set of indices of dominant channel taps of a wireless channel at time nT*_s_ *for an appropriately chosen ε. The channel is called effectively delay K-sparse in the duration of* $\left[n_{1}T_{s},\;n_{2}T_{s}\right]$ *if it satisfies* $K=K_{n}$ *and* $K=\left|K\right|\ll L$*, where* $n_{1}\leq n\leq n_{2}$ *and L is the total number of channel taps.*

For fast time-varying channels in wideband OFDM systems, it is known from Section 3.1 that, although the gain of the nonzero tap varies rapidly, we can still assume that its delay position remains unchanged within *M* consecutive OFDM symbols. When the wireless channel is effectively delay *K*-sparse during this period, according to definition 1, it can be determined that $\left|h_{n,l}\right|\approx 0,-N_{g}\leq n\leq N_{v}M-N_{g}-1,\;l\notin K$. Furthermore, from Equation (3), one can obtain $\left|\alpha_{l}^{\left(m\right)}\right|\approx 0,\;0\leq m\leq M-1,\;l\notin K$, which shows that the TATG vector $\alpha^{\left(m\right)}={\left[\alpha_{0}^{\left(m\right)},\;\ldots ,\;\alpha_{L-1}^{\left(m\right)}\right]}^{T}$ of the *m*-th OFDM symbol is also sparse. In summary, the TATG matrix $A=[\alpha^{\left(0\right)},\;\ldots ,\;\alpha^{\left(M-1\right)}]$ belongs to JSM-2. That is to say, each column in A has *K*-dominant elements, which appear at the same positions as the dominant elements of other columns.

### 4.2. The DCS Formulation

**Figure 4:**
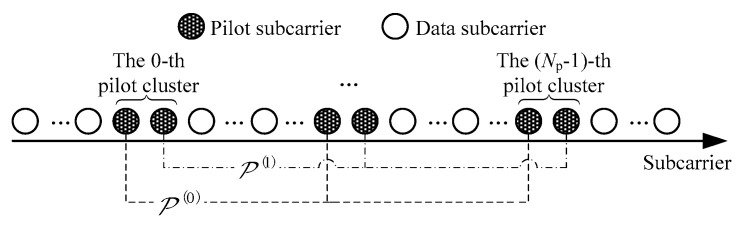
Pilot pattern.

The TATG matrix, A, can be estimated using the received pilots. The proposed pilot pattern is depicted in Figure 4, which is the same in each OFDM symbol. The pilot pattern contains $N_{p}$ pilot clusters, and each pilot cluster contains two adjacent pilot subcarriers with antipodal values (P,−P). The design of this kind of pilot cluster, called pilot ICI self-cancellation, can greatly reduce the interference of data on pilot subcarriers [16]. Since DCS is used to estimate A, we can save pilots by making $N_{p}<L$. Furthermore, we will determine the placement of $N_{p}$ pilot clusters by solving the optimization problem in Section 4.3 to ensure that A can be reconstructed with high probability.

The index set of all pilot subcarriers can be expressed as

(17) $$P=P^{\left(0\right)}\cup P^{\left(1\right)}$$

where $P^{\left(0\right)}$ and $P^{\left(1\right)}$ are sets containing the indices of the first pilot and the second pilot in all pilot clusters, respectively. Let $P^{\left(0\right)}=\{p_{k}|k=0,1,\cdots ,N_{p}-1\}$, and $P^{\left(1\right)}$ can be expressed as

(18) $$P^{\left(1\right)}=\{{p^{\prime }}_{k}|{p^{\prime }}_{k}={\left((p_{k}+1)\right)}_{N},\;k=0,\cdots ,N_{p}-1,\;p_{k}\in P^{\left(0\right)}\}.$$

Next, we construct the DCS framework for estimating the TATG matrix for *M* consecutive OFDM symbols.

For the *m*-th transmitted OFDM symbol, the pilot vectors $x_{p}^{\left(m,q\right)}$ are the subvectors extracting the elements of $x^{\left(m\right)}$ according to the indices in $P^{\left(q\right)}$, q=0,1. To achieve pilot ICI self-cancellation, a pair of antipodal values (P,−P) is modulated onto the two subcarriers in each pilot cluster, where P∈ℂ is an element of the modulation constellation set. Thus, $x_{p}^{\left(m,q\right)}$ can expressed as

(19) $$x_{p}^{\left(m,q\right)}={\left(-1\right)}^{q}\cdot P\cdot 1_{N_{p}},m=0,1,\ldots ,M-1,\;q=0,1$$

where $1_{N_{p}}$ is an $N_{p}\times 1$ vector with all elements as 1. From Equation (4), the corresponding pilot vectors of the *m*-th received OFDM symbol can be obtained as

(20) $$y_{p}^{\left(m,q\right)}=\mathrm{diag}\{x_{p}^{\left(m,q\right)}\}F_{p}^{\left(q\right)}\alpha^{\left(m\right)}+H_{p}^{\left(m,q\right)}x^{\left(m\right)}+w_{p}^{\left(m,q\right)},m=0,1,\ldots ,M-1,\;q=0,1$$

where $F_{p}^{\left(q\right)}\in {\mathbb{C}}^{N_{p}\times L}$ and $H_{p}^{\left(m,q\right)}\in {\mathbb{C}}^{N_{p}\times N}$ denote the submatrices extracting the rows of $F_{L}$ and ${\bar{H}}_{F}^{\left(m\right)}$ according to the indices in $P^{\left(q\right)}$, respectively. And $w_{p}^{\left(m,q\right)}\in {\mathbb{C}}^{N_{p}\times 1}$ denotes the subvector extracting the elements of $w^{\left(m\right)}$ according to the indices in $P^{\left(q\right)}$. From Equation (18), it can be derived that $F_{p}^{\left(1\right)}=F_{p}^{\left(0\right)}E$, where $E=\mathrm{diag}\{1,\;e^{-j\frac{2\pi }{N}},\;\cdots ,\;e^{-j\frac{2\pi }{N}(L-1)}\}$. The second term on the right-hand side of Equation (20) is the ICI item. Substituting Equation (19) into Equation (20), and then performing simple algebraic manipulations, we can obtain

(21) $$z^{\left(m\right)}=\Phi \alpha^{\left(m\right)}+\gamma^{\left(m\right)},m=0,1,\ldots ,M-1$$

where

(22) $$z^{\left(m\right)}=\sum_{q=0}^{1}\mathrm{diag}\{x_{p}^{\left(m,q\right)}{\})}^{-1}y_{p}^{\left(m,q\right)}=\frac{1}{P}(y_{p}^{\left(m,0\right)}-y_{p}^{\left(m,1\right)})$$

(23) $$\Phi =F_{p}^{\left(0\right)}+F_{p}^{\left(1\right)}=F_{p}^{\left(0\right)}(I_{L}+E)$$

(24) $$\begin{array}{l} \gamma^{\left(m\right)}=\sum_{q=0}^{1}\mathrm{diag}\{x_{p}^{\left(m,q\right)}{\})}^{-1}(H_{p}^{\left(m,q\right)}x^{\left(m\right)}+w_{p}^{\left(m,q\right)})\\ =\frac{1}{P}(H_{p}^{\left(m,0\right)}-H_{p}^{\left(m,1\right)})x^{\left(m\right)}+\frac{1}{P}(w_{p}^{\left(m,0\right)}-w_{p}^{\left(m,1\right)}). \end{array}$$

Considering *M* consecutive OFDM symbols, we define $Z≜[z^{\left(0\right)},\ldots ,z^{\left(M-1\right)}]$ and $\Gamma ≜[\gamma^{\left(0\right)},\ldots ,\gamma^{\left(M-1\right)}]$. From Equation (21), the DCS framework can be obtained as

(25) Z=ΦA+Γ

where $Z\in {\mathbb{C}}^{N_{p}\times M}$, $\Phi \in {\mathbb{C}}^{N_{p}\times L}$, $A\in {\mathbb{C}}^{L\times M}$, and $\Gamma \in {\mathbb{C}}^{N_{p}\times M}$ are the observation matrix, the common measurement matrix, the TATG matrix, and the interference-plus-noise matrix, respectively.

### 4.3. Position Optimization of Pilot Clusters

According to the DCS theory [20,22], although $N_{p}<L$, all columns in the TATG matrix, A, can be reconstructed jointly with high probability through the common measurement matrix, Φ, satisfying the restricted isometry property (RIP) [25] or the mutual incoherence property (MIP) [26]. Due to the large computational complexity for verifying the RIP of Φ, we consider its MIP. The coherence bound [26] of Φ is defined as

(26) $$\mu (\Phi )≜\underset{0\leq u\neq v\leq L-1}{\max}\frac{\left|\varphi_{u}^{H}\varphi_{v}\right|}{||\varphi_{u}|{|}_{2}\cdot ||\varphi_{v}|{|}_{2}}$$

where $\varphi_{m}$ and $\varphi_{n}$ are two arbitrary columns of Φ. The smaller the coherence bound μ(Φ), the more accurate the recovery of A. Substituting Equation (23) into Equation (26) and performing a simple mathematical derivation, we can obtain

(27) $$\mu (\Phi )=\underset{0\leq u\neq v\leq L-1}{\max}\frac{1}{N_{p}}\left|\sum_{k=0}^{N_{p}-1}e^{-j\frac{2\pi }{N}p_{k}(v-u)}\right|,p_{k}\in P^{\left(0\right)}.$$

From Equation (27), μ(Φ) is determined by $P^{\left(0\right)}$ or, equivalently, by P. For clarity of expression, μ(Φ) is represented as $\mu (\Phi (P^{\left(0\right)}))$. Here, we determine the placement of pilot clusters to minimize $\mu (\Phi (P^{\left(0\right)}))$. In other words, the optimized positions of pilot clusters can be obtained by solving the following optimization problem:

(28) $$\left\{\begin{array}{l} \underset{P^{\left(0\right)}\in S}{\min}\mu (\Phi (P^{\left(0\right)}))\\ s.t.\;2\leq {\left((p-p^{\prime })\right)}_{N}\leq N-2,\;\forall \;p,p^{\prime }\in P^{\left(0\right)} \end{array}\right.$$

where $S_{p}$ is a set consisting of all candidate sets of $P^{\left(0\right)}$, and the constraint condition is to ensure that the $N_{p}$ pilot clusters do not overlap each other. Solving the optimization problem (28) by an exhaustive search is unrealistic due to large computational complexity. The methods based on the genetic algorithm (GA) [27] and the estimation of distribution algorithm (EDA) [28] can quickly approximate the optimal solution of the optimization problem (28).

When reconstructing joint sparse signals, the sparsity adaptive matching pursuit algorithm for DCS (DCS-SAMP) [29] does not require a priori information on the sparsity, *K*, which is more suitable for practical implementation. Consequently, the DCS-SAMP algorithm is utilized to reconstruct the TATG matrix, A, in Equation (25), and we can obtain an estimate, $\hat{A}$, for A and the index set, $\hat{K}$, of the nonzero rows of $\hat{A}$.

## 5. Proposed Channel Estimation Method

The implementation of the proposed channel estimation method can be divided into the modeling stage and the data transmission stage.

The pseudocode of the modeling stage is listed in Algorithm 1. There are two tasks in the modeling stage, both of which can be completed offline during system design. On one hand, the positions of pilot clusters are optimized by solving the optimization problem (28), and then the common measurement matrix, Φ, in the DCS frame is determined. On the other hand, the transformation matrix, $V^{\left(m\right)}$, of the MS-CE-BEM is calculated.
**Algorithm 1.** Pseudocode of the modeling stage.**Input**:
 $N,\;N_{g}$
 $,\;L,\;M,\;N_{p}$ **Output**:
 $P_{\mathrm{opt}}$,
 Φ
 $,\;V^{\left(m\right)}$
 , 0≤m≤M−1
(1) Solve the optimization problem (28) by utilizing the EDA-based method, and the optimized $P^{\left(0\right)}$, denoted as $P_{\mathrm{opt}}^{\left(0\right)}$, is obtained.(2) Calculate the optimized P, denoted as $P_{\mathrm{opt}}$, according to Equations (17) and (18). $P^{\left(1\right)}=\{{p^{\prime }}_{k}|{p^{\prime }}_{k}={\left((p_{k}+1)\right)}_{N},\;k=0,\cdots ,N_{p}-1,\;p_{k}\in P^{\left(0\right)}\}$ $P=P^{\left(0\right)}\cup P^{\left(1\right)}$(3) Determine the measurement matrix Φ according to Equation (23). Extract $F_{p}^{\left(0\right)}$ from $F_{L}$ according to $P^{\left(0\right)}$. $\Phi =F_{p}^{\left(0\right)}(I_{L}+E)$(4) According to Equation (15), calculate the transformation matrix $V^{\left(m\right)}$, 0≤m≤M−1. for (m=0; m<M; m++)  $V^{\left(m\right)}=Q^{\left(m\right)}U_{MN_{v}}^{(c)}B{\left(DB\right)}^{†}U_{M}$ end for

Real-time processing is required in the data transmission stage. This stage is divided into four steps, and its pseudocode is listed in Algorithm 2. In step 1, the observation matrix, Z, in the DCS frame is constructed based on the received pilot subcarriers. The model parameter matrix estimation, $\hat{A}$, of the MS-CE-BEM is obtained using the DCS-SAMP algorithm in step 2. Next, in step 3, ${\hat{\alpha }}_{l}$ is extracted from matrix $\hat{A}$. Finally, the channel tap estimations, ${\hat{h}}_{l}^{\left(m\right)}$, in *M* OFDM symbols are obtained in step 4.
**Algorithm 2.** Pseudocode of the data transmission stage.**Input**: *P*, Φ
 $,\;y_{p}^{\left(m,0\right)}$
 $,\;y_{p}^{(m,1)}$
 $,\;V^{\left(m\right)}$
 , 0≤m≤M−1 **Output**:
 ${\hat{h}}_{l}^{\left(m\right)}$
 $,\;l\in \hat{K}$
 , 0≤m≤M−1
(1) Determine the observation matrix Z according to Equation (22). for (m=0; m<M; m++)  $z^{\left(m\right)}=\left(y_{p}^{\left(m,0\right)}-y_{p}^{\left(m,1\right)}\right)/P$ end for $Z=[z^{\left(0\right)},\ldots ,z^{\left(M-1\right)}]$ (2) Reconstruct the TATG matrix in Equation (25) by the DCS-SAMP algorithm to obtain its estimate $\hat{A}$ and the index set $\hat{K}$ of its nonzero rows.(3) Extract ${\hat{\alpha }}_{l}$ from $\hat{A}={\left[{\hat{\alpha }}_{0},\;\ldots ,\;{\hat{\alpha }}_{L-1}\right]}^{T}$ for $l\in \hat{K}$. (4) Replace $\alpha_{l}$ in Equation (14) with ${\hat{\alpha }}_{l}$, followed by computing the estimate of ${\tilde{h}}_{l}^{\left(m\right)}$, denoted as ${\hat{h}}_{l}^{\left(m\right)}$, where $l\in \hat{K}$ and 0≤m≤M−1. for (m=0; m<M; m++)  while ($l\in \hat{K}$)   ${\tilde{h}}_{l}^{\left(m\right)}=V^{\left(m\right)}\alpha_{l}$  end while end for

Here, we briefly discuss the computational complexity of the data transmission stage. In step 2, according to [29], the upper bound of the computational complexity of the DCS-SAMP algorithm is $O(M\left\lceil K/M\right\rceil N_{p}L)$, where K is the joint sparsity of $\left\{\alpha^{\left(m\right)}\right\}$. In step 4, the computational complexity is $O(KM^{2}N)$. The complexity of steps 1 and 3 is negligible compared to that of steps 2 and 4. Thus, the computational complexity of the data transmission stage has an upper bound of $O(M\left\lceil K/M\right\rceil N_{p}L+KM^{2}N)$ for the proposed channel estimation method.

## 6. Simulation Results and Discussion

In this section, we will evaluate the performance of the proposed method and conventional methods in terms of subcarrier utilization rate, channel estimation error, and system bit error rate (BER) using numerical simulations.

In the numerical simulations, the parameters of the OFDM system are listed in Table 1. The case of *f*_d_*T* = 0.2 corresponds to a transmission on a device at the movement speed of $v_{m}\approx 360$ km/h, which can meet the wireless communication demands of current high-speed trains. The simulation channel is the ITU-R vehicular A channel [30], which has six taps with Jake’s Doppler spectrum. The total power of the simulation channel is normalized to 1.

In the MS-CE-BEM, the number of OFDM symbols for joint channel estimation is M=8. With regard to Gibbs phenomenon suppression, there are three cases in the simulations, i.e., symmetric expansion, baseline tilting, and absence of suppression; in the three cases, the basis vector numbers of the MS-CE-BEM take the maximum values satisfying the LS algorithm condition, which are 15, 8, and 7, respectively. The minimum mean square error (MMSE) method is utilized for frequency domain equalization, and channel coding is not involved. Assume that all subcarriers are exploited to transmit data or pilot symbols. Since the total number of channel taps is not known in advance, it is assumed that *L* = *N*_g_.

### 6.1. Subcarrier Utilization Rate

Table 2 shows the pilot patterns utilized in the conventional single-symbol CE-BEM [12], the conventional MS-CE-BEM [16], and the proposed MS-CE-BEM based on DCS. In the frequency domain Kronecker delta (FDKD) pilot pattern, each pilot cluster needs at least five pilots, consisting of three observation pilots and two guard pilots. We define the subcarrier utilization rate as the ratio of the number of information-bearing data subcarriers to the total number of subcarriers. Obviously, the subcarrier utilization rate is proportional to the spectral efficiency when other conditions are the same. The numbers of pilots required for the three methods mentioned above, as well as their subcarrier utilization rates, are shown in Table 2.

As shown in Table 2, the proposed method, in terms of the subcarrier utilization rate, not only significantly surpasses the single-symbol CE-BEM, but also outperforms the conventional MS-CE-BEM. This is because the MS-CE-BEM exploits the temporal correlation of channel taps among OFDM symbols, which can save the pilot symbols. The proposed method further exploits the delay sparsity of the channel taps, and its subcarrier utilization rate is further improved.

### 6.2. Channel Estimation Performance

**Figure 5:**
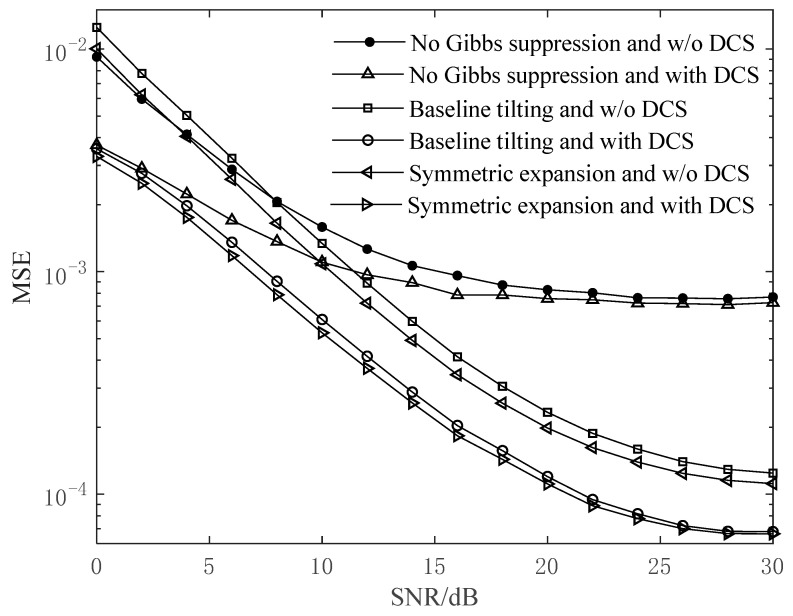
Channel estimation MSE performance with respect to SNR values at *f*_d_*T* = 0.2.

For the MS-CE-BEM, according to the previous description, there are three cases regarding the suppression of the Gibbs phenomenon and two cases depending on whether DCS is utilized or not. Among them, the DCS-based MS-CE-BEM with symmetric expansion is the one proposed in this paper. For the aforementioned six cases, the mean square error (MSE) of channel estimation with respect to signal-to-noise ratio (SNR) values at $f_{d}T=0.2$ is shown in Figure 5.

As can be seen from Figure 5, when the SNR is low, the MSE performance of channel estimation shows little difference in the three cases of symmetric expansion, baseline tilting, and absence of Gibbs phenomenon suppression. This is because both the symmetry expansion technique and the baseline tilting technique cannot reduce the noise level. However, the two techniques can effectively suppress the Gibbs phenomenon and reduce the model error of the MS-CE-BEM. As a result, with the increase in the SNR, the two techniques achieve a significant improvement in the MSE performance compared to the absence of Gibbs phenomenon suppression. On the other hand, Figure 5 shows the superior MSE performance of the methods with DCS over the ones without DCS, regardless of the SNR. The reason is that the DCS-based methods exploit the channel delay sparsity and the channel temporal correlation among multiple OFDM symbols, which can improve the recovery accuracy of the model input parameters contaminated by noise. It is noteworthy that, when the SNR is high and there is no suppression of the Gibbs phenomenon, the methods with DCS and without DCS show little difference in MSE performance. This is because the model error is the main factor leading to the degradation of MSE performance in this situation, and DCS cannot reduce it.

**Figure 6:**
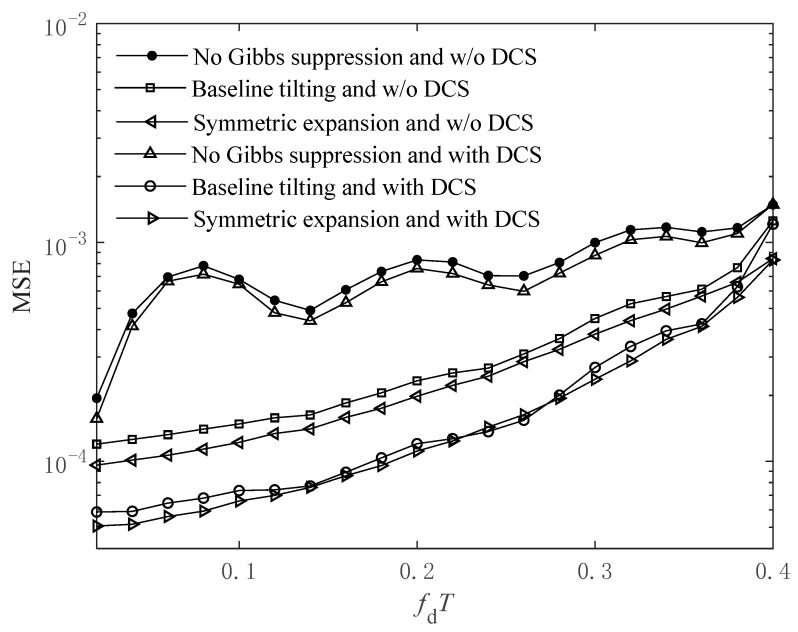
Channel estimation MSE performance with respect to *f*_d_*T* at SNR = 20 dB.

For the aforementioned six cases, the MSE performance comparison of channel estimation versus *f*_d_*T* at the SNR of 20 dB is shown in Figure 6.

From Figure 6, it can be seen that the MSE performance comparison of the six cases coincides with the observations in Figure 5. In addition, the MSE performance of the methods using the baseline tilting technique decreases rapidly with the increase in *f*_d_*T* when *f*_d_*T* > 0.36. However, the MSE performances of the methods using the symmetric expansion technique only have relatively stable decreases until *f*_d_*T* = 0.4. This shows that the symmetric expansion technique has a higher upper limit of *f*_d_*T* than the baseline tilting technique and can be applied to faster time-varying channels, which coincides with the observation in Figure 3.

### 6.3. System BER Performance

**Figure 7:**
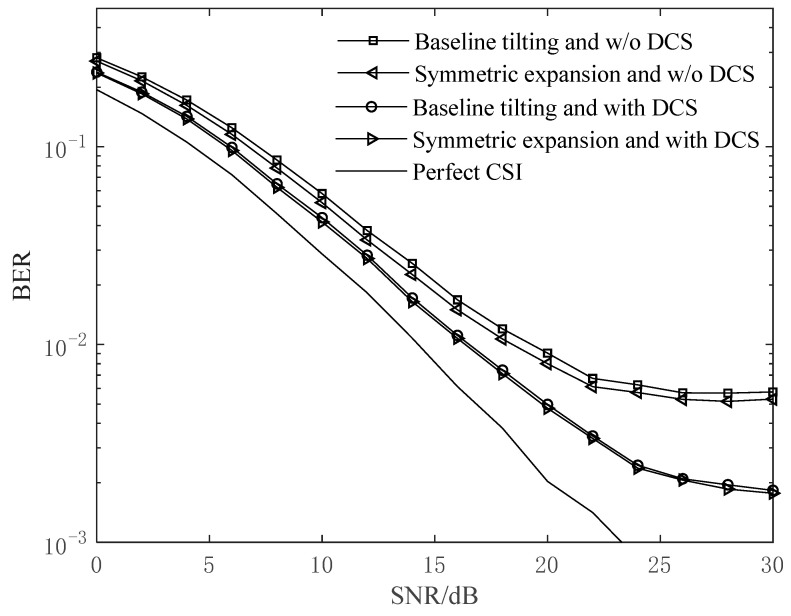
BER performance comparison of the five cases at *f*_d_*T* = 0.2.

With *f*_d_*T* being 0.2, Figure 7 depicts the system BER performance of the MS-CE-BEM in different cases, as well as that with perfect CSI.

From Figure 7, we can find that, when the SNR is low, the BER performance curves of the various cases show little difference due to the high noise level. When the SNR increases gradually, the methods with DCS achieve significant improvements in the BER performance compared with that without DCS. To achieve a BER of $6\times 10^{-3}$, for example, the methods with DCS have an advantage of about 5 dB in terms of required SNRs compared to those without DCS. With the SNR being greater than 24 dB, the error floor begins to appear for the methods without DCS, while the error floor does not appear until the SNR is greater than 30 dB for the methods with DCS.

## 7. Conclusions

To address the two issues of large channel estimation errors and low spectrum utilization of OFDM systems in high-mobility scenarios, we have explored the temporal correlation of time-varying channels within multiple OFDM symbols and the joint sparsity of the TATG matrix in wideband OFDM systems, and proposed the time-varying channel estimation method based on DCS and the MS-CE-BEM. By integrating pilot ICI self-cancellation, the optimization of the sparse pilot pattern, and symmetric extension technique, the proposed method demonstrates good performance under certain mobility. The simulation results show that, compared with the existing MS-CE-BEM without DCS, the proposed method significantly improves the subcarrier utilization rate, the channel estimation MSE performance, and the system BER performance. In the next step of the research, we will explore how the number of OFDM symbols within the model can adapt to system mobility. On this basis, we will generalize the mathematical framework of the proposed method.

## Figures and Tables

**Table 1:** OFDM system parameters.

Parameter Types	Parameter Values
Carrier frequency (*f*_c_)	5.8 GHz
Bandwidth (*B*_W_)	10 MHz
Symbol modulation mode of pilot and data	QPSK
Number of subcarriers (*N*)	1024
Length of CP (*N*_g_)	10 MHz

**Table 2:** The pilot patterns and the subcarrier utilization rates.

	The Conventional Single-Symbol CE-BEM [12]	The Conventional MS-CE-BEM [16]	The Proposed MS-CE-BEM Based on DCS
Pilot pattern	FDKD	See [16]	See Section 4.2 and Section 4.3
Number of pilot clusters	*N*_g_ (64)	*N*_g_ (64)	*N*_p_ (20)
Number of pilots per pilot cluster	5	2	2
Number of pilots per OFDM symbol	320	128	40
Subcarrier utilization rate (%)	68.75	87.50	96.09

## Data Availability

Data are contained within the article.
